# A systematic review of the pharmacokinetic and pharmacodynamic interactions of herbal medicine with warfarin

**DOI:** 10.1371/journal.pone.0182794

**Published:** 2017-08-10

**Authors:** Songie Choi, Dal-Seok Oh, Ui Min Jerng

**Affiliations:** 1 The K-herb Research Center, Korea Institute of Oriental Medicine, Daejeon, South Korea; 2 Clinical Research Division, Korea Institute of Oriental Medicine, Daejeon, South Korea; Universita degli Studi di Napoli Federico II, ITALY

## Abstract

**Objectives:**

The aim of this study was to systematically review data regarding pharmacokinetic (PK)-pharmacodynamic (PD) parameters from randomized controlled trials relating to interactions between herbal medicines and warfarin.

**Methods:**

Three electronic databases were searched to identify relevant trials. Two reviewers independently performed the study selection and data extraction. The risk of bias and reporting quality were also assessed independently by two reviewers using the Cochrane risk of bias tool and the consolidated standards of reporting trials (CONSORT). Outcomes were measured for all reported PK-PD parameters and adverse events.

**Results:**

Nine randomized controlled trials met our inclusion criteria. Most of the included studies were unclear regarding the risk of bias and had a low quality of methodology. Using CONSORT, the reporting percentages for the articles ranged from 36.5% to 61.5% and the mean percentage for all articles was 45.6%. St John’s wort and echinacea affected the PK parameters of warfarin. Ginseng, ginger, garlic, and cranberry had no significant effect on the PK parameters. American ginseng altered the PD parameters of warfarin. St John’s wort, ginseng, Korea red ginseng, ginkgo, ginger, garlic, aged garlic, and echincea did not significantly alter the PD parameters. Studies of ginkgo and cranberry showed conflicting results on the PK parameters and PD parameters, respectively. The incidence of adverse events in all trials was low and no major adverse events were reported.

**Conclusions:**

It was difficult to determine whether ten herbal medicines had significant effects on the PK-PD parameters of warfarin. Low quality of evidence, different compounds within and different compositions of the herbs, and methodological limitations of the crossover study, which is a clinical study in which subjects receive a sequence of different interventions, made it difficult to form conclusions. Additional studies that remedy these vulnerabilities are necessary to verify these results.

## Introduction

Warfarin is the most common oral anticoagulant used for treating or preventing thromboembolic disorders. It has a narrow range between therapeutic and toxic doses, suggesting that warfarin should be administered after calculating the optimal dose.

Patients taking warfarin should also be aware of its interaction with other drugs and foods, including herbal medicines, because the concomitant use of these agents might alter the metabolism and action of warfarin, necessitating an adjustment to the dose of warfarin for its safe and effective administration [1, 2]. Close monitoring of the anticoagulant effect of warfarin is recommended through the international normalized ratio (INR) in clinical practice [1–3].

Herbal medicines are often used by patients receiving anticoagulants. Nearly 40% of patients with cardiovascular disease have used complementary and alternative medicine, including herbal medicine, concomitantly with their prescribed medications [4]. Those who used herbal medicine for health management perceived herbal medicine to be helpful for their cardiac condition [5]. In fact, some herbs, such as ginger, ginkgo, and garlic, have antiplatelet and anticoagulant activity [6–11]. However, the mechanism of action of herbal medicines is difficult to study *in vitro* and *in vivo* because these medicines comprise complex mixtures of various compounds [12], which may simultaneously exhibit multiple physiological activities. Therefore, patients taking herbal medicine with warfarin are more likely to be exposed to potential herb-drug interactions [13].

Previous studies have revealed some of the mechanisms of interaction of warfarin with herbal medicines via clinical reports [2, 14]. Each herb has a different chemical composition, hindering generalizations about herb-warfarin interactions. Although narrative reviews on herb-warfarin interactions are available [1, 5], no study has systematically reviewed them based on changes in pharmacokinetic (PK)-pharmacodynamic (PD) parameters.

The aim of this article was to systematically review clinical data, including PK-PD parameters, from randomized controlled trials (RCTs) and to discuss interactions between herbal medicines and warfarin.

## Methods

### Sources of information and search strategies

Clinical trials were searched for and retrieved from core electronic databases, including PubMed, EMBASE, and CINAHL. The last search of the databases was performed in December 2015.

Search terms consisted of text terms and controlled vocabulary, such as medical subject headings (MeSH). Three types of search terms were used: warfarin-related terms, herb-related terms, and interaction-related terms. Article type or study design-related terms were not included in the search terms. The search strategy for PubMed is stated below. The search terms for the two other databases were similar.

#1 Warfarin [MeSH Terms]
#2 Warfarin [Title/Abstract]
#3 1–2 /or
#4 Dietary supplementations [MeSH Terms]
#5 Dietary supplement* [Title/Abstract]
#6 Plant, medicinal [MeSH Terms]
#7 Phytotherapy [MeSH Terms]
#8 Medicine, traditional [MeSH Terms]
#9 Pharmacognosy [MeSH Terms]
#10 Plant extracts [MeSH Terms]
#11 Ethnobotany [MeSH Terms]
#12 Ethnopharmacology [MeSH Terms]
#13 Diet, Food, and Nutrition [MeSH Terms]
#14 Plant* [Title/Abstract]
#15 Herb* [Title/Abstract]
#16 4-15/or
#17 Drug interactions [MeSH Terms]
#18 Interaction* [Title/Abstract]
#19 17-18/or
#20 3 AND 16 AND 19

### Study selection

Two reviewers (SIC and UMJ) reviewed the titles and abstracts of the studies retrieved from the electronic searches to identify studies that met the inclusion criteria. Disagreements were resolved by discussion between the two reviewers or consultation with a third reviewer (DSO). No language restriction was applied. The inclusion criteria were as follows:

- **Type of study**. All relevant RCTs that reported interactions between herbal medicines and warfarin were included.
- **Type of participant**. Studies that evaluated subjects who received herbal medicine concomitantly with warfarin were included.
- **Type of intervention**. Trials using warfarin alone or warfarin with placebo drug versus warfarin with herbal medicine were included. An herb was defined as a product or an extract originating from a single botanical source. The definition of herb included raw or manufactured single or complex medicinal plants, plant extracts, and dietary supplements. However, single or synthesized substances from plant material were excluded.
- **Type of outcome measures**. Studies that measured more than one PK or PD parameter for herb-warfarin interactions were included. Because the inhibition of the metabolism of S-warfarin is clinically more important than the inhibition of the metabolism of R-warfarin, the PK of R-warfarin were not investigated in this study [15, 16]. The PK parameters included time to maximum plasma concentration (T_max_), maximum plasma concentration at steady state (C_max_), apparent volume of distribution after extra vascular administration (V/F), fraction of total drug unbound in plasma (f_u_), terminal half-life (T_1/2_), apparent plasma clearance of drug after extra vascular administration (CL/F), and area under the plasma concentration-time curve from zero to infinity (AUC_inf_). The PD parameters included all outcomes that reflected the biochemical and physiological effects of warfarin on the human body.

### Data extraction and quality assessment

Data were extracted from the titles and abstracts of the searched studies independently by two reviewers. The study selection and data extraction used standard eligibility inclusion criteria as determined by two reviewers. The quality of methodology in all included studies was independently assessed according to the Cochrane Collaboration’s seven criteria: 1) random sequence generation, 2) allocation concealment, 3) blinding of participants and personnel, 4) blinding of outcome assessment, 5) incomplete outcome data, 6) selective reporting, and 7) other bias (defined as baseline data comparability). For each domain, the evaluation was denoted as low risk, high risk, or unclear risk, according to the description of the methods used in each study.

We also assessed the reporting quality of all included studies based on the Consolidated Standards of Reporting Trials (CONSORT) [17]. We used the CONSORT 2010 checklist and the extension of the CONSORT statement simultaneously for trials of herbal medicinal interventions. The Consort 2010 checklist is the latest version for assessing reporting quality. The elaborated CONSORT statements for trials of herbal interventions enhance the checklist items regarding the relevance to trials of herbal interventions [18, 19].

### Data analysis

Study design and herbal medicines were analyzed among the included studies. Results of PK or PD parameters and type and proportion of adverse events in concurrent use of herbal medicine and warfarin groups were compared with those in warfarin alone groups to identify whether herbal medicine significantly affected the PK or PD parameters of warfarin. Quantitative data synthesis was planned in a meta-analysis when the study design, type of herbal medicine, and outcomes of the included studies were homogeneous; otherwise, we suggested results in a narrative synthesis without meta-analysis [20].

## Results

### Description of included studies

**Fig 1:**
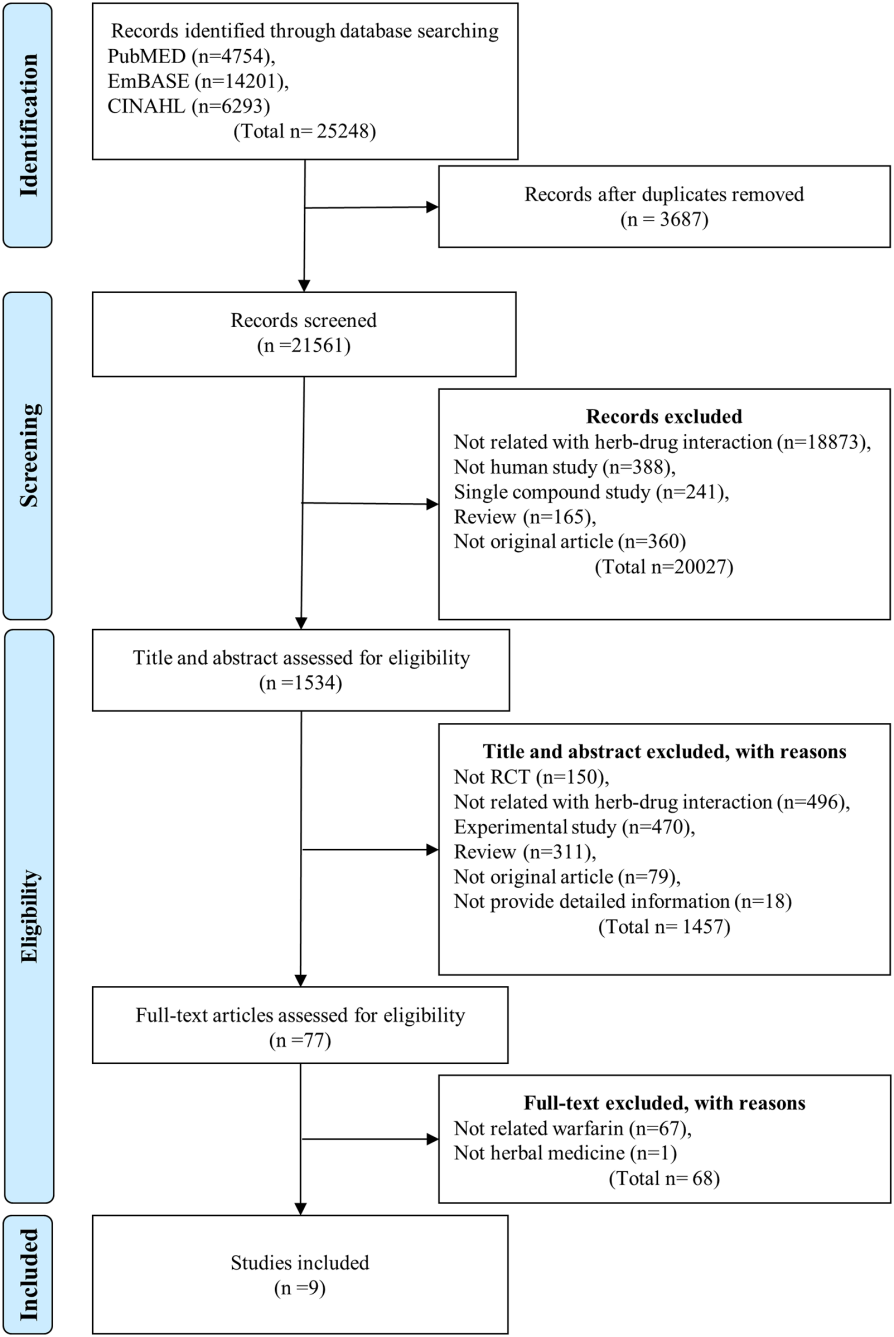
PRISMA flow diagram for selecting related articles.

The search generated a total of 4437 potentially relevant studies; 295 duplicate and 4065 irrelevant studies were excluded by screening the titles and abstracts. Of the remaining studies, 77 full-text articles were reviewed and 9 studies [21–29] met our eligibility criteria. The PRISMA diagram of the search process and study selection is presented in Fig 1.

Of these trials, three [23–25] were conducted in the United States, four [21, 22, 26, 28] were conducted in Australia, and one [27] was conducted in Korea. One trial [29] was conducted in China and published in Chinese. Four studies [21, 22, 26, 28] had a three-way cross-over randomized design and compared two different herbal medicine plus warfarin groups with a warfarin alone group in healthy subjects. Three studies [23, 25, 29] used a placebo-controlled parallel design and compared the concomitant administration of herbal medicine and warfarin with the concomitant administration of placebo and warfarin. Two studies [24, 27] used a double-blind crossover design. The study sample sizes ranged from 7 to 48 and a total of 160 subjects were involved in the nine trials. Thirty participants were reported to have dropped out of the nine studies. The key data from the included RCTs are summarized in Table 1.

**Table 1:** Characteristics of the included studies (n = 9).

1^st^author, Country	Jiang [21], Australia	Jiang [22], Australia	Yuan [23], United States	Li [24], United States	Macan [25], United States	Abdul [26], Australia	Lee [27], Republic of Korea	Abdul [28], Australia	Zhou [29], China
**Study design (RCT)**	Open-label, three-way crossover	Open-label, three-way crossover	Double-blind, placebo-controlled	Double-blind, crossover	Double-blind, placebo-controlled	Open-label, three- way treatment crossover	Double-blind, crossover	Open-label, three-way treatment crossover	Two-way treatment, placebo-controlled crossover
**The number of subjects(The number of male subjects)**	12(12)	12(12)	20(9)	7(7)	48(30)	12(12)	25(4)	12(12)	12(6)
**Type of subjects**	Healthy volunteers	Healthy volunteers	Healthy volunteers	Atrial fibrillation patients	Deep vein thrombosis, cerebro-vascular accident, thrombosis, valvular heart disease, atrial fibrillation, or prosthetic heart valves patients	Healthy volunteers	Cardiac valve replacement patients	Healthy volunteers	Healthy volunteers
**Age**	20–40	20–36	Treatment group: 30.2 (Mean) Placebo group: 24.3 (Mean)	68.8 (Mean)	56 (Mean)	18–34	-	18–34	19–24
**Intervention in treatment group (+warfarin)**	St John's wort Ginseng	Ginkgo Ginger	America Ginseng	Cranberry	Aged Garlic	Cranberry Garlic	Korea red ginseng	Echinacea	Ginkgo
**Intervention in control group (+warfarin)**	None	None	Placebo	Placebo	Placebo	None	Placebo	Placebo	Placebo
**Treatment Period** (1^st^/2^nd^ period, if cross-over)	2weeks/1week	1week/1week	4weeks	1week/1week	12weeks	2weeks/1week	6weeks	2week/1week	5weeks
**Washout Period**	2weeks	2weeks	Not applicable	1week	Not applicable	2weeks	3weeks	2weeks	-
**The number of observed adverse events**	3	1	0	Not reported	0	4	Not reported	0	0
**Results reported**	St John's wort induced the metabolism of warfarin on human with a subsequent effect on INR/ Korean ginseng had little effect or warfarin metabolism	No affect the pharmacokinetics or pharmacodynamics of either S-warfarin and coagulation status	Reduces the anticoagulant effect of warfarin	No any significant interaction	No increase in the incidence of hemorrhages	Cranberry juice extract for 2 weeks significantly increased the sensitivity/ Garlic did not have significant effects on platelet aggregation	Not enhance the anticoagulation effect	No affected warfarin pharmacodynamics, platelet aggregation or baseline clotting status	No effects on the pharmacodynamics of single dose warfarin

Abbreviations: INR, International Normalized Ratios.

### Herbal medicine

Nine herbal medicines were identified in the included studies: *Panax ginseng*, *Panax quinquefolius*, *Allium sativum*, *Gingko biloba*, *Vaccinium macrocarpon*, *Hypericum perforatum*, *Echinacea angustifolia*, *Echinacea purpurea*, and *Zingiber officinale*. Ginseng [21, 23, 27] was administered in three studies, but three ginsengs that have different scientific names were used: Korean ginseng root (*Panax ginseng*) [21], American ginseng root (*Panax quinquefolius*) [23], and Korea red ginseng (steamed *Panax ginseng*) [27]. Garlic (*Allium sativum*)was administered in two studies, but each study used garlic manufactured with a different process [25, 26]. One study [25] used an aged garlic product that was made by soaking raw garlic in ethanol, whereas the other study [26] used an enteric-coated garlic tablet. Gingko (*Gingko biloba*) [22, 29] and cranberry (*Vaccinium macrocarpon*) [24, 26] were used in two studies and St John’s wort (*Hypericum perforatum*) [21], echinacea (Mixture of *Echinacea angustifolia* and *Echinacea purpurea*) [28], and ginger (*Zingiber officinale*) [22] were administered in one study. In addition, policosanol [28] was mentioned in one study. However, policosanol is a complex mixture of fatty alcohols derived from sugar cane wax and was not included in the inclusion criteria. Extraction and formulation method, composition, and bioanalytical data regarding the herbal preparations from the included RCTs are summarized Table 2.

**Table 2:** Herbal preparations of the included studies (n = 9).

1^st^author	Jiang [21]		Jiang [22]		Yuan [23]	Li [24]	Macan [25]	Abdul [26]		Lee [27]	Abdul [28]	Zhou [29]
**Material (Scientific name)**	St John's wort (*Hypericum perforatum)*	Panax Ginseng (*Panax ginseng)*	Ginkgo (*Gingko bilba)*	Ginger (*Zingiber officinale)*	America Ginseng (*Panax quinquefolius)*	Cranberry (*Vaccinium macrocarpon)*	Garlic (*Allium sativum)*	Cranberry (*Vaccinium macrocarpon)*	Garlic (*Allium sativum)*	Red ginseng (*Panax ginseng)*	Echinacea *(Echinacea angustifolia* and *Echinacea purpurea)*	Ginkgo (*Gingko biloba)*
**Preprocessing of material**	ND	ND	ND	ND	Grinding	ND	Slicing	ND	ND	Steaming	ND	ND
**Type of extract**	Dry extract	Dry extract	Dry extract	ND	None	Juice	Long-term maceration	ND	ND	Decoction	ND	ND
**Solvent**	ND	ND	ND	ND	None	ND	Aqueous ethanol	ND	ND	Water	ND	ND
**Formulation type**	Tablet	Capsule	Tablet	Capsule	Capsule	Packaged liquid	Solid	Capsule	Tablet	Powder	Tablet	Tablet
**Commerical product**	Yes	Yes	Yes	Yes	No	Yes	Yes	Yes	Yes	ND	Yes	ND
**Constituents & Qualty Control**	1 g of *Hyperricum perforatum*, 0.825 mg of hypericin and 12.5 mg of hyperforin	0.5 g of *Panax ginseng* root and 8.93 mg of ginsenosides as ginsenoside R_g1_	2 g of *Ginkgo biloba* leaf, 9.6 mg of ginkgo flavonglycosides, 2.4 mg of ginkgolides and bilobalide	0.4 g of ginger rhizome powder	5.19% of total ginsenoside (Ginsenoside R_b1_: 1.93%; R_b2_: 0.20%; R_c_: 0.61%; R_d_: 0.42%; R_e_: 1.68%; R_g1_: 0.35%)	Food-grade quality for human consumption Packaging	Containing 305 g/L of extracted solids Concentration of S-allycysteine, active compound: 1.47 g/L Pharmaceutical Good Manufacturing Practices	High concentration of anthocytanins and quercetins Physical stability test	2000 mg of fresh garlic bulb equivalent to 3.71 mg of allicin per tablet Assay for Allicin-releasing characteristics	100 mg/g of saponins and more than 60% of solid powder	600mg of *Echinacea angustilfolia* roots and 675 mg of *Echinacea purpurea* root, 5.75 mg of total alkamides per tablet	9.6 mg of total flavonol glycosides, 2.4 mg of terpene lactones

Abbreviations: ND, not described

### Risk of bias assessment

**Fig 2:**
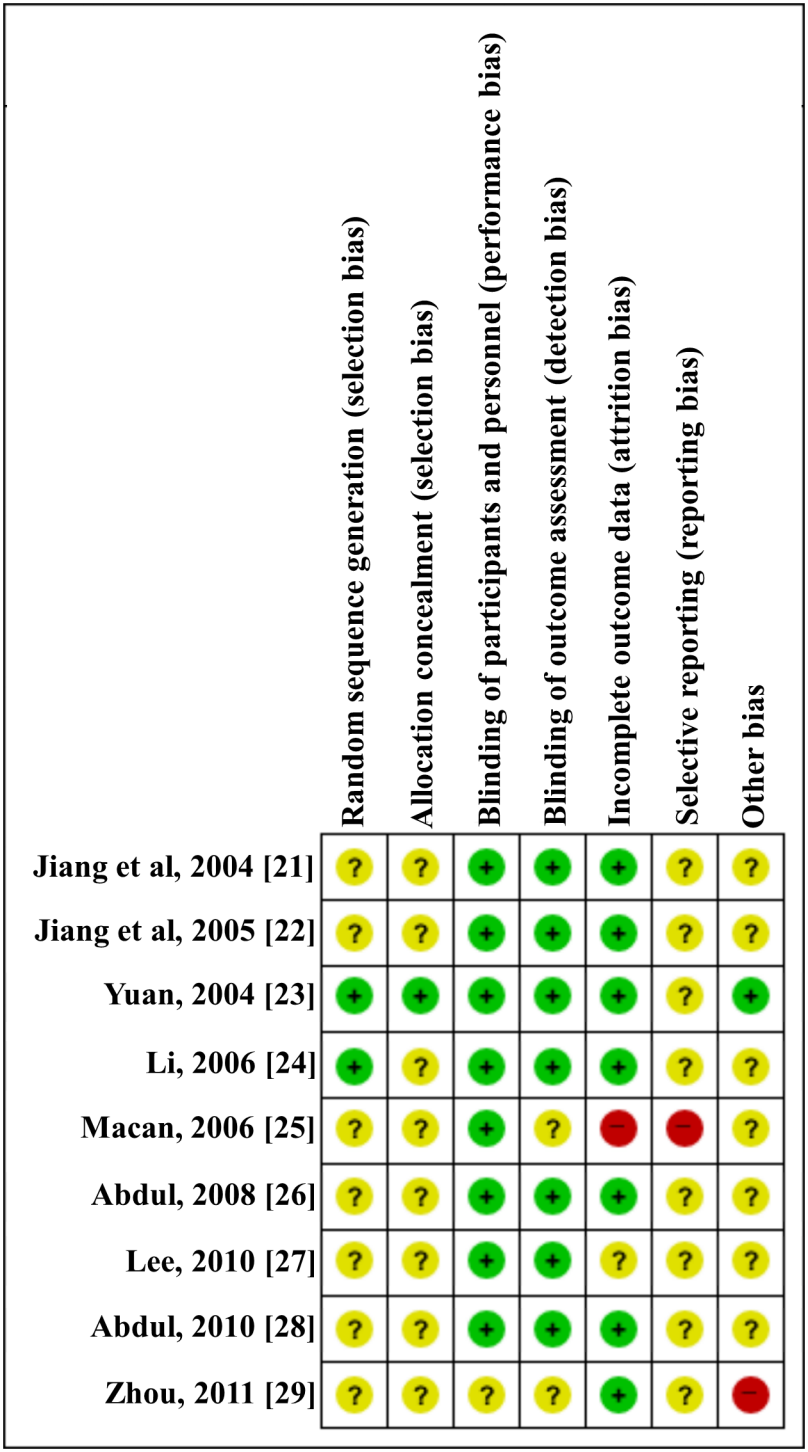
Risk of bias summary. Review of authors' judgments about each risk of bias item for all nine included studies. Plus (+) marked circle, Low risk of bias; Question (?) marked circle, Unclear risk of bias; Minus (-) marked circle, High risk of bias.

**Fig 3:**
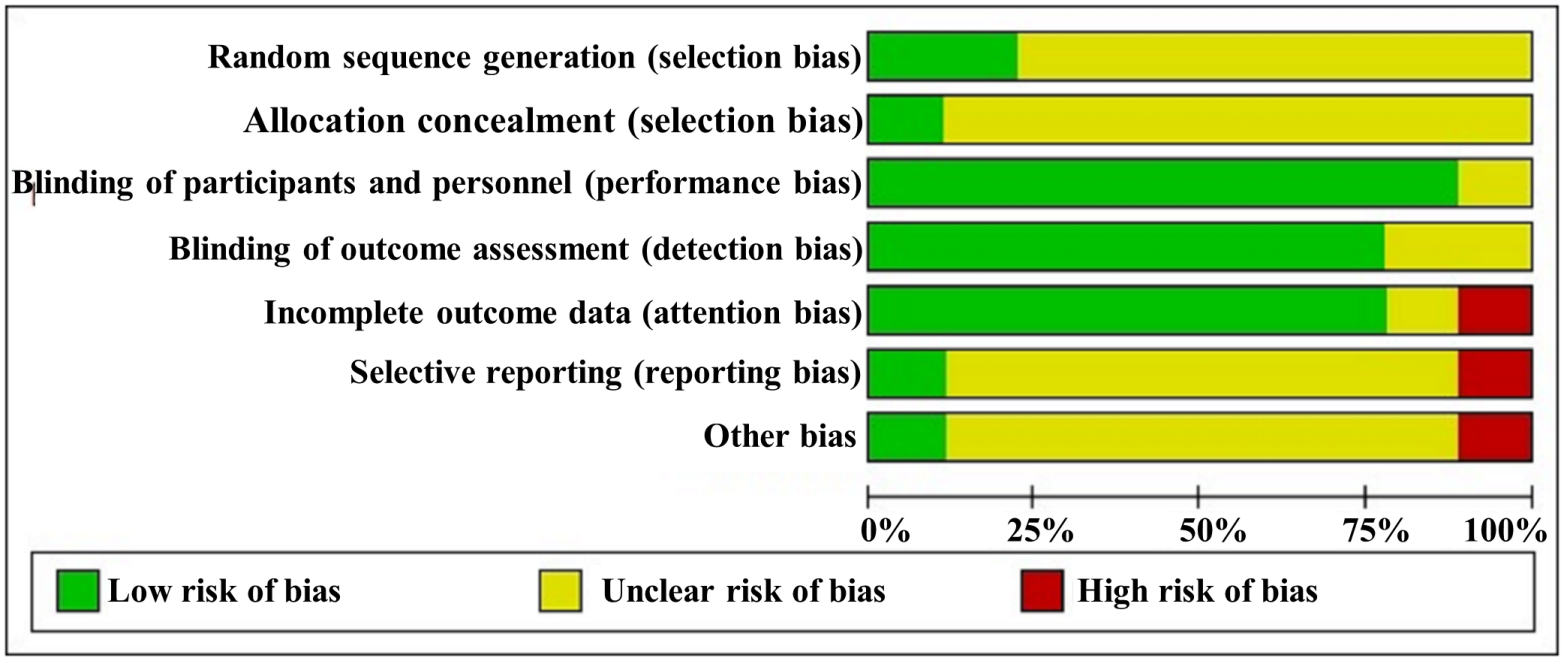
Risk of bias graph. Review of authors' judgments about each risk of bias item presented as percentages across all included studies.

No study had a low risk of bias in all seven domains. For random sequence generation, two [23] of the nine studies (22%) used a random table, whereas the other studies (78%) did not report a specific method of random sequence generation. For allocation concealment, one study [23, 24] (11%) used an opaque envelope method, whereas the other studies (89%) did not report any information about concealment. For blinding, four studies [23–25, 27] (44%) used a double-blinding method by blinding participants and researchers and four studies [21, 22, 26, 28] had an open-label design. One study [29] did not provide information about blinding. For incomplete outcome data, seven trials [21–24, 26, 28, 29] reported detailed information regarding attrition by describing the number and reasons for withdrawal. For selective outcome reporting, only one study [26] presented the clinical trial identifier number, whereas the other trials did not report registration information. Therefore, we could not compare the protocols and trial reports. Information for other risks of bias was not reported in the studies, except for one study [29] that was at high risk. The risk of bias assessment information is presented in Figs 2 and 3.

### Reporting quality

Based on the two CONSORT statements, the reporting percentage for each of the articles ranged from 36.5% to 61.5% and the mean percentage for all articles was 45.6%. All RCTs described the eligibility criteria, participant flow, and interpretation of the results. No trials provided information about the qualitative testing of interventions, periods of recruitment, or follow up. Detailed results are presented in the S1 Table.

Because the two CONSORT statements were developed with the aim of evaluating and reporting quality for parallel design and two treatment groups, they may be insufficient to evaluate and reflect the characteristics of the crossover design. However, most of the items in the CONSORT checklist apply to all trial designs because they reflect the characteristics of RCTs rather than those of parallel design. Therefore, the CONSORT statements were used to assess the reporting quality of all the included trials.

### Outcomes

There are many different methods of measuring outcome parameters. Because the aim of this review was to assess herb-warfarin interactions, various types of PK-PD parameters were evaluated (S2 Table).

Five trials [21, 22, 26, 28, 29] reported PK data based on the absorption, distribution, metabolism, or elimination of warfarin when herbal medicine was co-administered with warfarin. There were three herbs that affected the PK of warfarin in healthy subjects. St John’s wort [21] increased S-warfarin clearance and reduced R-warfarin clearance. Echinacea [28] increased the apparent clearance of warfarin. One study [29] reported that ginkgo significantly increased C_max_, AUC_inf_, and T_1/2_ and decreased the CL/F of warfarin, whereas another study [22] reported that ginkgo did not markedly change the PK parameters of warfarin. There were no significant changes in the PK parameters of warfarin when ginseng [21], ginger [22], garlic [26], and cranberry [26] were co-administered. Garlic and cranberry did not affect the S-warfarin clearance in subjects with different genotypes of cytochrome P450 2C9 (CYP2C9) [26]. Whereas garlic increased the half maximal effective concentration (EC_50_) of S-warfarin in the subjects with the CC genotype of vitamin K epoxide reductase complex (VKORC1), cranberry decreased the EC_50_ of S-warfarin in the subjects with the CT or TT genotype of VKORC1 [26].

The PD parameters of warfarin were reported when ten herbal medicines, including ginkgo, ginger, ginseng, St John’s wort, echinacea, cranberry, Korean red ginseng, American ginseng, garlic, and aged garlic were co-administered with warfarin. The AUC of INR for time to treatment was used in six studies [21–23, 26, 28, 29], INR max [26, 28] was evaluated in two studies, and INR change was reviewed in two studies [24, 27]. Five studies [21, 22, 26, 28, 29] used area under concentration-time curves until the last concentration observation (AUC_obs_) as an outcome measure. Platelet aggregation [21, 22], peak INR change [23], vitamin K intake [23], prothrombin time [29], and incidence of hemorrhage [25] were also included as PD parameters. The results of two studies [23, 26] indicated that co-administration of herbal medicine altered the PD parameters of warfarin. The results of one study indicated that cranberry significantly increased the area under the INR-time curve when administered with warfarin in healthy subjects [26]. However, the results of another study indicated that cranberry did not markedly change INR values in patients with atrial fibrillation [24]. American ginseng also reduced the anticoagulant effect of warfarin [23]. There were no significant changes in the PD parameters of warfarin when St John’s wort [21], ginseng [21], Korea red ginseng [27], ginkgo [22, 29], ginger [22], garlic [26], aged garlic [25], and echinacea [28] were co-administered with warfarin.

### Adverse events

All trials evaluated adverse events (AEs). As shown in Table 1, AEs were reported for three studies and none of the events was major. Among these events, one study [22] reported that one subject experienced gastrointestinal side effects, including constipation, during the first two days of ginkgo pre-treatment and mild diarrhea during the first two days of ginger pre-treatment. One study [21] reported that three subjects experienced changes in sleeping habits during St John’s wort treatment and one study [26] reported rashes in two subjects using cranberry-warfarin. In studies including garlic, one subject had evidence of nasal bleeding and one subject reported lip dryness.

## Discussion

In this review, the interaction between ten herbs and warfarin, as indicated by changes in the PK and PD parameters of warfarin, was analyzed based on published evidence. We assessed the methodological quality of RCTs using the Cochrane risk of bias tool and CONSORT and the results of quality assessments were reflected in the interpretation of this study. Two herbs (St John’s wort and echinacea) affected the PK parameters of warfarin, whereas four herbs (ginseng, ginger, garlic, and cranberry) did not. There were conflicting results as to whether ginkgo affected the PK parameters of warfarin. American ginseng changed the PD parameters of warfarin, but eight herbal medicines (St John’s wort, ginseng, Korea red ginseng, ginkgo, ginger, garlic, aged garlic, and echinacea) did not. There were mixed results as to whether cranberry changed the PD parameters of warfarin. There was a low risk of AEs after co-administration of herbs and warfarin. However, most of the included studies had low reporting quality and a crossover design that was unsuitable for meta-analysis. There were also inconsistent results from several studies that used the same herbal medicine.

The use of herbal medicine is rapidly expanding and many reports have raised concerns about possible herb-drug interactions. Herb-drug interactions may be categorized as either PK or PD interactions. PK interactions include changes in absorption, distribution, metabolism, and elimination [30]. PD interactions result from synergistic, additive, or antagonistic effects of herbs when co-administered with drugs. Well-aligned PK-PD data provides information regarding clinical efficacy and safety outcomes and guides the selection of doses and dosing schedules for clinical trials [31].

All included studies investigated the PD interactions of warfarin with herbal medicines, but different parameters were used in each study. Only four studies reported PK interaction parameters between an herbal medicine and warfarin, whereas the five remaining studies did not measure PK parameters. There were inconsistent results among those that used the same herbal medicine. Two studies that evaluated the interaction between cranberry and warfarin reported contradictory PD effects, possibly because one study [26] investigated healthy subjects and the other [24] included patients with atrial fibrillation. The results of two studies that assessed the PK-PD interactions between warfarin and ginkgo also differed. One study [22] reported no significant differences in PK-PD parameters, whereas another [29] observed that ginkgo had limited effects on PK parameters. These studies used different types of clinical designs and subject conditions. Three studies [21, 23, 27] investigated the interaction between ginseng and warfarin and the results differed according to the type of ginseng used (Korean ginseng root (*Panax ginseng*), American ginseng root (*Panax quinquefolius*), or Korea red ginseng (steamed *Panax ginseng*)). Korean ginseng and American ginseng have different ginsenoside profiles [32] and Korea red ginseng contains converted ginsenosides transformed from the ginsenosides in fresh ginseng [33]. The heterogeneous composition of compounds among the three types of ginseng might have led to different results.

Several case reports have pointed to the risk associated with concomitant herb and warfarin use. There were two case reports of an increased INR after co-administration of warfarin with cranberry juice [34, 35]. Interactions between ginseng and warfarin were also mentioned in one case report [36]. Two case reports suggested that a warfarin-St John’s wort interaction was associated with a change in INR [37, 38]. These relevant case reports indicated a potential herb-warfarin interaction, but it was difficult to identify a causal relationship. Suspected herb-warfarin interactions are primarily limited to anecdotal case reports. In addition, these case reports did not provide sufficient information about the patients’ medical records and compounding factors may have existed, such as administration of other medications, dietary supplements, foods, or alcohol intake. Some studies pointed out such limitations, that is, that case reports often result in misleading conclusions for multiple reasons [39, 40]. There have been previous experiments and clinical studies of platelet aggregation caused by herbs. Several studies have suggested that herbal constituents may affect PK-PD and alter the anticoagulant and platelet aggregation effects of warfarin [41, 42].

Other studies have shown conflicting results as to whether herb-warfarin interactions were associated with increased risks. Garlic and ginger are known potent inhibitors of platelet aggregation [43]. One review article reported that spontaneous bleeding occurred during the concurrent use of warfarin and these herbs. Conversely, some cases did not indicate a significant inhibition of platelet function [44]. One *in vitro* study suggested that gingko contributed to the altered platelet aggregation [45]. In contrast, a clinical study confirmed that gingko did not change platelet aggregation. Furthermore, it was difficult to determine whether the combined use of warfarin and herbs led to increased platelet aggregation [46].

Warfarin is predominantly metabolized via CYP2C9 and changes in CYP2C9 may significantly alter the PK-PD parameters of warfarin [35, 39]. However, an *in vivo* study indicated that echinacea did not significantly affect the metabolism of drugs metabolized by CYP2C9 [47]. Another *in vivo* study reported that gingko induced CYP enzyme activity in a dose-dependent manner, but did not cause hepatic damage [48]. Two clinical trials also evaluated the effects of ginkgo in healthy volunteers and the results indicated that warfarin concentrations did not significantly change with concomitant administration of ginkgo [22, 29]. These results implied that concerns regarding increased hemorrhagic complications resulting from an herb-warfarin interaction were unfounded. This study also indicated that herbal medicine might not lead to a clinically significant change in the PK-PD parameters of warfarin. Furthermore, herbal medicine did not significantly alter the anticoagulant effects of warfarin and no severe AEs were reported.

There were four limitations of the current review. First, the rationale for determining the washout period in each study was lacking. Several studies adopted a crossover design, which has been used by many researchers to investigate potential drug-drug interactions. Because a crossover trial carries the risk of a carry-over effect, trials with a crossover design should use a sufficient washout period. Otherwise, the effect of the first period treatment may persist into the subsequent period [49]. The average washout period in seven studies was two weeks and one study did not mention the washout period. To minimize the carry-over effect, the washout period in a crossover study should be at least five times the half-life of the drug [50]. The mean range of T_1/2_ was 29.2 to 38.7 hours for the control group and 27.2 to 76.6 hours for the experimental group. The washout period was calculated to be 6–8 days for the control group and 6–16 days for the experimental group based on these results. These results included studies for which it was difficult to determine if the washout period was sufficient.

Second, clinical data are lacking in order to provide synthesized evidence for herb-drug interactions from crossover trials. We attempted to conduct a meta-analysis using five trials that assessed the effects of herbs combined with warfarin versus warfarin alone or warfarin combined with placebo. A meta-analysis for crossover trials can be conducted if one of the following three measurements is available: 1) individual subject data, 2) the mean and standard deviation (or standard error) of the subject-specific differences between the experimental group and control group, 3) the mean difference and variables from a paired t-test (28). However, no studies were reported these data. Therefore, it was impossible to synthesize the data into a meta-analysis.

Third, the reporting quality of the included studies was poor based on CONSORT 2010 and the extension of the CONSORT statement for trials of herbal medicine interventions. This CONSORT extension enhances the checklist items regarding the relevance of herbal interventions to trials [19]. Several studies did not describe some of the items in this elaborated statement. For example, some studies autonomously prepared the intervention drug or herbal medicine without a quality control, making it difficult to report the characteristics of the herbal product, including the concentration of the extraction solvent, the method of the authentication of raw material, fingerprinting, and standardization. The majority of studies also did not discuss randomization, including sequence generation, allocation, and concealment. The four crossover design studies did not report how the treatment group and control group were crossed. Therefore, the response rate for each article was less than 50%.

Finally, all included RCTs focused on changes in the PK-PD parameters of warfarin and not those of the herbal medicine. Each single herb has a variety of biochemical compounds and the composition of components in the herb could vary upon cultivation, delivery, and product manufacturing conditions. These uncertainties and complexities make it difficult to determine standards for the PK-PD parameters of herbal medicines. Therefore, this study could not include PK-PD evaluation of herbal medicines themselves and did not reflect the purpose of the administration and intention for use of the herbal medicine.

Further studies evaluating interactions between warfarin and herbal medicines should avoid the limitations mentioned in this study. The details of the information for a trial should be clearly described and fully reported. The crossover design is a common study design, but is inappropriate for obtaining valid evidence through meta-analysis. We recommend using high-quality RCTs to confirm herb-warfarin interactions. In addition, to ensure the quality of the clinical trials, randomization and allocation concealment procedures should be performed to minimize bias.

Although there are some limitations, to our knowledge, this study was the first systematic review of the clinical outcomes and PK-PD effects of herb-warfarin interactions. We deliberately selected related studies after searching a wide range of databases and this analysis was based on available clinical trials that evaluated herb-warfarin interactions.

## Conclusions

It was difficult to decide whether ten herbal medicines significantly affected the PK-PD parameters of warfarin. Low quality of evidence, herbal uncertainties and complexities of different compounds and their compositions, and methodological limitations of the crossover study made it difficult to form conclusions. Further studies with an appropriate study design and reporting quality are necessary to verify herb-warfarin interactions.

## Supporting information

S1 Table Reporting quality of RCTs based on the consolidated standards of reporting trials (CONSORT). (DOCX)

S2 Table Pharmacokinetic and pharmacodynamic parameters of the included studies (n = 6). (DOCX)

S1 Checklist PRISMA 2009 checklist. (DOC)
