# Supplementary material for: A systematic review of the pharmacokinetic and pharmacodynamic interactions of herbal medicine with warfarin
Source: PLoS One. 2017 Aug 10;12(8):e0182794. doi: 10.1371/journal.pone.0182794 (PMC5552262; doi:10.1371/journal.pone.0182794)
Supplement: S1 Table — (DOCX) [file pone.0182794.s001.docx]

**S1 Table.** Reporting quality of RCTs based on the consolidated standards of reporting trials (CONSORT).

| **Section/Topic** | **Item No** | **Checklist item** | **Jiang**  **[21]** | **Jiang**  **[22]** | **Yuan**  **[23]** | **Li**  **[24]** | **Macan**  **[25]** | **Abdul**  **[26]** | **Lee**  **[27]** | **Abdul**  **[28]** | **Zhou**  **[29]** |
| --- | --- | --- | --- | --- | --- | --- | --- | --- | --- | --- | --- |
| **Title and abstract** | |  |  |  |  |  |  |  |  |  |  |
|  | 1a | Identification as a randomised trial in the title | N | N | N | N | N | N | N | N | N |
|  | 1b | Structured summary of trial design, methods, results, and conclusions (for specific guidance see CONSORT for abstracts) | Y | Y | Y | Y | Y | Y | N | Y | N |
| **Introduction** | |  |  |  |  |  |  |  |  |  |  |
| Background and objectives | 2a | Scientific background and explanation of rationale | Y | Y | Y | Y | Y | Y | Y | Y | Y |
|  | 2b | Specific objectives or hypotheses | Y | Y | Y | Y | Y | Y | Y | Y | Y |
| **Methods** | |  |  |  |  |  |  |  |  |  |  |
| Trial design | 3a | Description of trial design (such as parallel, factorial) including allocation ratio | Y | Y | Y | Y | Y | Y | Y | Y | Y |
|  | 3b | Important changes to methods after trial commencement (such as eligibility criteria), with reasons | N/A | N/A | N/A | N/A | N/A | N/A | N/A | N/A | N/A |
| Participants | 4a | Eligibility criteria for participants | Y | Y | Y | Y | Y | Y | Y | Y | Y |
|  | 4b | Settings and locations where the data were collected | N | N | Y | N | N | N | N | N | N |
| Interventions | 5 | The interventions for each group with sufficient details to allow replication, including how and when they were actually administered | Un | Un | Y | Y | Y | Un | Y | Un | Un |
|  | 5A (Herbal medicinal product name) | 1. The Latin binomial name together with botanical authority and family name for each herbal ingredient common name(s) should also be included. | Y | Y | Y | Y | N | Y | N | Y | N |
|  |  | 2. The proprietary product name (i.e., brand name) or the extract name (e.g., EGb-761) and the name of the manufacturer of the product. | Y | Y | Y | N | Y | Y | Y | Y | Y |
|  |  | 3. Whether the product used is authorized (licensed registered) in the country in which the study was conducted. | Y | Y | N | N | Y | N | N | Y | N |
|  | 5B (Characteristics of the herbal product) | 1. The part(s) of plant used to produce the product or extract. | Y | Y | Y | N | Y | N | N | Y | Y |
|  |  | 2. The type of product used (e.g., raw [fresh or dry], extract). | Y | Y | Y | Y | Y | Y | Y | N | Y |
|  |  | 3. The type and concentration of extraction solvent used (e.g., 80% ethanol, 100% H2O, 90% glycerine, etc.) and the ratio of herbal drug to extract (e.g., 2 to 1). | N | N | N | N | Y | N | Y | N | N |
|  |  | 4. The method of authentication of raw material (i.e., how done and by whom) and the lot number of the raw material. State if a voucher specimen (i.e., retention sample) was retained and, if so, where it is kept or deposited, and the reference number. | Un | Un | Y | N | N | N | N | N | N |
|  | 5C(Dosage regimen and quantitative description) | 1. The dosage of the product, the duration of administration, and how these were determined. | Y | Y | Y | Un | Y | Y | Y | Y | Y |
|  |  | 2. The content (e.g., as weight, concentration; may be given as range where appropriate) of all quantified herbal product constituents, both native and added, per dosage unit form. Added materials, such as binders, fillers, and other excipients (e.g., 17% maltodextrin, 3% silicon dioxide per capsule), should also be listed. | Y | Y | Y | N | Y | Y | Y | Y | Y |
|  |  | 3. For standardized products, the quantity of active/marker constituents per dosage unit form. | Y | Y | N/A | N | Y | Y | N/A | Y | Y |
|  | 5D(Qualitative testing) | 1. Product’s chemical fingerprint and methods used (equipment and chemical reference standards) and who performed the chemical analysis (e.g., the name of the laboratory used); whether a sample of the product (i.e., retention sample) was retained and if so, where it is kept or deposited. | N | N | N | N | N | Y | N | N | N |
|  |  | 2. Description of any special testing/purity testing (e.g., heavy metal or other contaminant testing) undertaken, which unwanted components were removed and how (i.e., methods). | N | N | N | N | N | N | N | N | N |
|  |  | 3. Standardization: what to standardize (e.g., which chemical components of the product) and how (e.g., chemical processes or biological/functional measures of activity). | N | N | N | N | N | N | N | N | N |
|  | 5E(Placebo/controlgroup | The rationale for the type of control/placebo used. | N | N | Y | Y | N | N/A | Y | N/A | Y |
|  | 5F | A description of the practitioners (e.g., training and practice experience) that are a part of the intervention. | N | N | N | N | N | N | N | N | N |
| Outcomes | 6a | Completely defined pre-specified primary and secondary outcome measures, including how and when they were assessed | Y | Y | Y | Y | N | Y | Y | Y | Y |
|  | 6b | Any changes to trial outcomes after the trial commenced, with reasons | N/A | N/A | N/A | N/A | N/A | N/A | N/A | N/A | N/A |
| Sample size | 7a | How sample size was determined | N | Y | N | N | N | N | N | N | N |
|  | 7b | When applicable, explanation of any interim analyses and stopping guidelines | N/A | N/A | N/A | N/A | N/A | N/A | N/A | N/A | N/A |
| **Randomisation** | |  |  |  |  |  |  |  |  |  |  |
| Sequence generation | 8a | Method used to generate the random allocation sequence | N | N | Y | N | N | N | N | N | N |
|  | 8b | Type of randomisation; details of any restriction (such as blocking and block size) | N | N | Y | N | N | N | N | N | N |
| Allocation concealment mechanism | 9 | Mechanism used to implement the random allocation sequence (such as sequentially numbered containers), describing any steps taken to conceal the sequence until interventions were assigned | N | N | Y | N | N | N | N | N | N |
| Implementation | 10 | Who generated the random allocation sequence, who enrolled participants, and who assigned participants to interventions | N | N | Y | N | N | N | N | N | N |
| Blinding | 11a | If done, who was blinded after assignment to interventions (for example, participants, care providers, those assessing outcomes) and how | N/A | N/A | Y | N | N | N/A | N | N/A | N |
|  | 11b | If relevant, description of the similarity of interventions | N/A  (Open-label) | N/A  (Open-label) | Y | Y | N | N/A  (unblinding) | Y | N/A  (unblinding) | N |
| Statistical methods | 12a | Statistical methods used to compare groups for primary and secondary outcomes | Y | Y | Y | Y | Y | Y | Y | Y | Un |
|  | 12b | Methods for additional analyses, such as subgroup analyses and adjusted analyses | N/A | N/A | N/A | N/A | N/A | Y | N/A | N/A | N/A |
| **Results** | |  |  |  |  |  |  |  |  |  |  |
| Participant flow (a diagram is strongly recommended) | 13a | For each group, the numbers of participants who were randomly assigned, received intended treatment, and were analysed for the primary outcome | Y | Y | Y | Y | Y | Y | Y | Y | Y |
|  | 13b | For each group, losses and exclusions after randomisation, together with reasons | N/A | N/A | N/A | N/A | N | Y | Y | N | N/A |
| Recruitment | 14a | Dates defining the periods of recruitment and follow-up | N | N | N | N | N | N | N | N | N |
|  | 14b | Why the trial ended or was stopped | N/A | N/A | N/A | N/A | N/A | N/A | N/A | N/A | N/A |
| Baseline data | 15 | A table showing baseline demographic and clinical characteristics for each group | N | N | N | Y | Y | N | Y | N | N |
| Numbers analysed | 16 | For each group, number of participants (denominator) included in each analysis and whether the analysis was by original assigned groups | Y | Y | Y | N | y | Un | Un | N | N |
| Outcomes and estimation | 17a | For each primary and secondary outcome, results for each group, and the estimated effect size and its precision (such as 95% confidence interval) | Y | Y | Y | Y | Y | Y | Y | Y | Y |
|  | 17b | For binary outcomes, presentation of both absolute and relative effect sizes is recommended | N | Y | Y | N/A | Y | Y | N/A | y | y |
| Ancillary analyses | 18 | Results of any other analyses performed, including subgroup analyses and adjusted analyses, distinguishing pre-specified from exploratory | N/A | N/A | N/A | N/A | N/A | Y | N/A | N/A | N/A |
| Harms | 19 | All important harms or unintended effects in each group (for specific guidance see CONSORT for harms) | Y | Y | Y | Y | Y | Y | N | Y | Y |
| **Discussion** | |  |  |  |  |  |  |  |  |  |  |
| Limitations | 20 | Trial limitations, addressing sources of potential bias, imprecision, and, if relevant, multiplicity of analyses | Y | Y | Y | Y | Y | Y | N | Y | Y |
| Generalisability | 21 | Generalizability (external validity) of the trial findings. | Y | Y | Y | Y | Y | Y | Y | N | Y |
| Interpretation | 22 | Interpretation consistent with results, balancing benefits and harms, and considering other relevant evidence | Y | Y | Y | Y | Y | Y | Y | Y | Y |
| **Other information** | |  |  |  |  |  |  |  |  |  |  |
| Registration  Protocol | 23 | Registration number and name of trial registry | N | N | N | N | N | Y | N | N | N |
|  | 24 | Where the full trial protocol can be accessed, if available | N | N | N | N | N | Y | N | N | N |
| Funding | 25 | Interpretation consistent with results, balancing benefits and harms, and considering other relevant evidence | Y | Y | Y | Y | N | Y | Y | Y | N |

Abbreviations: Y, Yes; N, NO; N/A, not applicable; Un, unclear.
