# Supplementary material for: A systematic review of the pharmacokinetic and pharmacodynamic interactions of herbal medicine with warfarin
Source: PLoS One. 2017 Aug 10;12(8):e0182794. doi: 10.1371/journal.pone.0182794 (PMC5552262; doi:10.1371/journal.pone.0182794)
Supplement: S2 Table — (DOCX) [file pone.0182794.s002.docx]

**S2 Table.** Pharmacokinetic and Pharmacodynamic parameters of the included studies (n = 9).

| **Author** | **Treatment** | **PK** | | | | | | | | | **PD** |
| --- | --- | --- | --- | --- | --- | --- | --- | --- | --- | --- | --- |
|  |  | **Absorption** | | **Distribution** | | **Metabolism** | | **Elimination** | | **Observed exposure** | **AUC_0-168_ of INR** |
|  |  | T_max_ (hr) | C_max_ (㎍/mL) | V/F(L/kg) | Fu(%) | CYP | VKORC1 | T_1/2_ (h) | CL/F(mL/h) | AUC_0-∞_(㎍/mL·h) |  |
| Jiang [21] | Warfarin + St John's wort | 1.26±0.46  (0.97-1.55) | 1.82±0.34  (1.6-2.0) | 0.13±0.03  (0.11-0.15) | 0.36±0.01  (0.30-0.43) | - | - | 25.1±4.3  (22.4-27.9) | 270±44  (241-297) | 47.7±8.3  (42.4-53.0) | 88.3±30.7  (68.8-107.8) |
|  | Warfarin + Ginseng | 1.30±0.55  (0.95-1.65) | 1.93±0.31  (1.7-2.1) | 0.13±0.03  (0.11-0.15) | 0.39±0.16  (0.29-0.49) | - | - | 29.2±5.2  (25.9-32.4) | 220±29  (201-238) | 57.8±7.4  (53.1-62.5) | 111.1±43.1  (83.9-138.7) |
|  | Warfarin | 1.29±0.51  (0.97-1.62) | 1.89±0.26  (1.7-2.0) | 0.12±0.03  (0.11-0.14) | 0.34±0.11  (0.27-0.41) | - | - | 31.7±4.5  (28.8-34.5) | 198±38  (174-223) | 65.4±13.8  (56.6-74.1) | 111.0±49.3  (79.6-142.3) |
| Jiang [22] | Warfarin + Ginkgo | 1.4  (0.9-1.9) | 1.8  (1.5-2.0) | 0.12  (0.11-0.13) | - | - | - | 35.1  (30.9-39.3) | 200  (173-227) | 65.8  (55.1-76.5) | 121  (77-165) |
|  | Warfarin + Ginger | 1.6  (1.1-2.1) | 1.7  (1.5-2.0) | 0.12  (0.11-0.13) | - | - | - | 35.7  (30.0-41.3) | 201  (171-231) | 66.0  (54.0-78.1) | 125  (91-160) |
|  | Warfarin | 2.1  (1.4-2.8) | 1.7  (1.4-2.0) | 0.12  (0.11-0.14) | - | - | - | 35.8  (31.1-40.3) | 189  (167-210) | 68.0  (60.8-75.3) | 124(90-158) |
| Yuan [23] | Warfarin + America Ginseng | - | - | - | - | - | - | - | - | - | - |
|  | Warfarin + Placebo | - | - | - | - | - | - | - | - | - | - |
| Li [24] | Warfarin + Cranberry | - | - | - | - | - | - | - | - | - | - |
|  | Warfarin + Placebo | - | - | - | - | - | - | - | - | - | - |
| Macan [25] | Warfarin + Aged garlic | - | - | - | - | - | - | - | - | - | - |
|  | Warfarin + Placebo | - | - | - | - | - | - | - | - | - | - |
| Abdul [26] | Warfarin + Cranberry | 1.9  (1.1-2.7) | 1.7  (1.5-2.0) | - | 0.010  (0.007-0.014) | No change for CYP2C9 | Mutant showed  lower EC_50_ values | 34.9  (31.0-38.8) | 204  (183-224) | 63.0  (57.3-68.8) | 119.2  (97.1-141.4) |
|  | Warfarin + Garlic | 1.3  (1.1-1.6) | 1.9  (1.5-2.2) | - | 0.011  (0.008-0.014) | No change for CYP2C9 | Wild type showed  higher EC_50_ values | 41.2  (35.1-47.3) | 202  (175-229) | 65.5  (55.3-65.6) | 100.3  (70.9-129.8) |
|  | Warfarin | 1.4  (1.1-1.6) | 1.8  (1.7-1.9) | - | 0.010  (0.007-0.013) | No change for CYP2C9 | Wild type showed  higher EC_50_ values | 38.6  (35.7-41.5) | 213  (193-232) | 60.3  (54.5-66.1) | 96.0  (72.1-119.8) |
| Lee [27] | Warfarin + Korean red ginseng | - | - | - | - | - | - | - | - | - | - |
|  | Warfarin + Placebo | - | - | - | - | - | - | - | - | - | - |
| Abdul [28] | Warfarin + Echinacea | 1.9  (1.4-2.4) | 1.3  (1.1-1.43) | 0.23  (0.16-0.30) | 0.01  (0.01-0.02) | - | - | 36.5  (34.1-38.8) | 289.7  (218.2-361.1) | 49.0  (40.0-57.9) | 55.2  (42.8-67.6) |
|  | Warfarin | 1.7  (1.1-2.2) | 1.3  (1.1-1.6) | 0.21  (0.16-0.27) | 0.01  (0.01-0.01) | - | - | 38.6  (34.3-43.0) | 267.3  (198.4-336.3) | 53.9  (42.9-64.8) | 52.7  (38.3-67.2) |
| Zhou [29] | Warfarin + Ginkgo | 1 | 0.7±0.1 | 15.2±4.3 (L) | - | - | - | 76.6±28.4 | 150±30 | 36.4±5.5 | 275.5±28.3 |
|  | Warfarin + Placebo | 0.725 | 0.6±0.1 | 12.7±3.3 (L) | - | - | - | 29.2±5.2 | 170±20 | 32.4±5.2 | 255.1±34.2 |

The variables are presented Mean±Standard deviation (95% Confidence interval); Abbreviations: PK, pharmacokinetics; PD, pharmacodynamics; T_max_, time to maximum plasma concentration; C_max_, maximum plasma concentration; V/F, volume of distribution; FU, fraction unbound; CYP, cytochrome p450 gene; CYP2C9, cytochrome p4502C9 gene; VKORC1, vitamin K epoxide reductase subunit 1; t_1/2_, elimination half-life; CL/F, apparent clearance; AUC, area under plasma concentration-time curve; AUC_0-168_ of INR, The area under the INR-time curve until 168h; EC_50_,  half maximal effective concentration; h, hours.
